# Identifications of potential therapeutic targets and drugs in angiotensin II-induced hypertension: Erratum

**DOI:** 10.1097/MD.0000000000009590

**Published:** 2018-01-05

In the article, “Identifications of potential therapeutic targets and drugs in angiotensin II-induced hypertension”,^[1]^ which appeared in Volume 96, Issue 46 of *Medicine*, the authors would like to note that Xiaoli Wu and Ruihua Fan contributed equally to the article and are co-first authors.
